# Uterine rupture revisited: Predisposing factors, clinical features, management and outcomes from a tertiary care center in Turkey

**DOI:** 10.12669/pjms.293.3625

**Published:** 2013

**Authors:** Abdulkadir Turgut, Ali Ozler, Mehmet Siddik Evsen, Hatice Ender Soydinc, Neval Yaman Goruk, Talip Karacor, Talip Gul

**Affiliations:** 1 Abdulkadir Turgut, MD, Department of Obstetrics and Gynecology, Dicle University School of Medicine, Diyarbakir, Turkey.; 2 Ali Ozler, MD, Department of Obstetrics and Gynecology, Dicle University School of Medicine, Diyarbakir, Turkey.; 3 Mehmet Siddik Evsen, MD, Department of Obstetrics and Gynecology, Dicle University School of Medicine, Diyarbakir, Turkey.; 4 Hatice Ender Soydinc, MD, Department of Obstetrics and Gynecology, Dicle University School of Medicine, Diyarbakir, Turkey.; 5 Neval Yaman Goruk, MD, Department of Obstetrics and Gynecology, Dicle University School of Medicine, Diyarbakir, Turkey.; 6 Talip Karacor, MD, Department of Obstetrics and Gynecology, Dicle University School of Medicine, Diyarbakir, Turkey.; 7 Talip Gul, MD, Department of Obstetrics and Gynecology, Dicle University School of Medicine, Diyarbakir, Turkey.

**Keywords:** Cesarean section, Perinatal outcome, Uterine rupture

## Abstract

***Objective:*** To determine the predisposing factors, modes of clinical presentation, management modalities and fetomaternal outcomes of uterine rupture cases at a tertiary care center in Turkey.

***Methodology:*** A 14-year retrospective analysis of 61 gravid (>20 weeks of gestation) uterine rupture cases between January 1998 to March 2012 was carried out.

***Results:*** The incidence of ruptured uteri was calculated to be 0.116%. Persistence for vaginal delivery after cesarean was the most common cause of uterine rupture (31.1%). Ablatio placenta was the most common co-existent obstetric pathology (4.9%). Bleeding was the main symptom at presentation (44.3%) and complete type of uterine rupture (93.4%) was more likely to occur. Isthmus was the most vulnerable part of uterus (39.3%) for rupture. The longer the interval between rupture and surgical intervention, the longer the duration of hospitalization was. Older patients with increased number of previous pregnancies were likely to have longer hospitalization periods.

***Conclusion:*** Rupture of gravid uterus brings about potentially hazardous risks. Regular antenatal care, hospital deliveries and vigilance during labor with quick referral to a well-equipped center may reduce the incidence of this condition.

## INTRODUCTION

Uterine rupture (UR) is a serious, life-threatening event that may cause peripartum hysterectomy, hemorrhage, shock, and even maternal and newborn mortality. Good antenatal care and advanced management of labour may aid in decreasing the incidence of UR. However, UR still appears to be a relatively common and serious obstetric catastrophe especially in developing countries.^1^^-^^3^ Immediate complications like anemia, urinary bladder rupture or shock and long-term complications such as infertility, foot drop or vesicovaginal fistula may be encountered due to UR.^4^^,^^5^

Previous cesarean section (CS) incision or other uterine scars, uterine anomalies, grand multiparity, tumours, use of oxytocin, placenta percreta, and fetal anomalies are postulated risk factors for UR.^4^ Prevalence of UR varies between 1:250 and 1:5000 deliveries.^3^^,^^6^ A complete UR involves the entire uterine wall leading to a direct connection between the peritoneal space and the uterine cavity, whereas a cover of visceral peritoneum or the broad ligament is left over the uterus in case of an incomplete UR.^4^ The underlying factors for UR include a poor referral system, non-attendance to antenatal care, delay in seeking medical care and delay of essential interventions.^5^ Low socioeconomic status, the delivery of babies >3.5 kg, HIV positivity and history of previous cesarian section are other postulated risk factors for UR. Oxytocin stimulation and previous uterine scars are assumed to be the direct causes of UR in developed countries, while obstructed labour is the main culprit in developing countries.^5^ Maternofetal outcomes of UR vary from country to country depending on the availability and quality of health facilities.

The aim of this study was to review the risk factors and causes of UR and deﬁne the modes of clinical presentation, complications, management, maternal and fetal outcome.

## METHODOLOGY

***Study design:*** A 14-year retrospective analysis of gravid (>20 weeks of gestation) UR case records from January 1998 to March 2012 was performed at the obstetrics and gynecology department of a tertiary care center. Approval of local Institutional Review Board had been obtained. Patients were assessed in terms of demographics such as age, parity, gestational age, obstetric history, mode of presentation, the use of uterine stimulant, the course of labour, clinical features, type and site of rupture and operative treatment, hospital stay and fetomaternal outcome.

In this series, complete UR was defined either as a full-thickness uterine wall defect accompanied with acute maternal bleeding that calls for operative intervention. Cases of uterine dehiscence or other partial defects of uterine wall were termed as incomplete UR.

***Statistical analysis:*** Data were analyzed using the Statistical Package for Social Sciences (SPSS) software version 19.0 for Windows (SPSS Inc., Chicago, IL). Parametric tests were applied to data of normal distribution and non-parametric tests were applied to data of questionably normal distribution. Pearson Chi Square and Mann–Whitney U-tests were used to compare independent groups. To calculate correlation coefficients Kendall's tau b was used. Data are expressed as mean±SD or median (interquartile range), as appropriate. Statistical significance was assumed for p<0.05.

## RESULTS

A total of 61 UR cases consisting of 57 (93.4%) complete and 4 (6.6%) incomplete cases have been identified during the 14-year period of the study. In this period, there were a total of 52,398 deliveries of which 61 patients had rupture of the gravid uterus giving a ratio of 1: 858 deliveries and the incidence of ruptured uteri was calculated to be 0.116%. Six patients delivered via vaginal route at home and reffered to our clinic due to excessive vaginal bleeding. Three of them had a previous CS history and tried to deliver via vaginally and one of them had a Kristeller maneuver trial at home. Uterine rupture detected in these patients during the operation performed for intraabdominal hemorrhage. In addition 10 patient reffered to our clinic from a city hospital due to excessive vaginal bleeding, accompanying pathologies (2 placenta percreta, 1 placenta accrete) that are lately diagnosed intraoperatively because of the vaginal delivery trial of the patients at home. The maternal demographic characteristics are displayed in Table-I. The mean maternal age was 32 (20-45) years, and the mean parity was 4.4 (0-11). The cesarean delivery was performed in 27 (44.3%) patients and 34 (55.7%) had given birth via vaginal route. The most common obstetric pathologies accompanying UR were ablatio placenta (4.9%), placenta previa percreata (3.3%), placenta previa accreata (1.6%) and uterine didelphys (1.6%). The most common complaint at initial admission was vaginal bleeding followed by hemodynamic instability defined as systolic blood pressure <90 mm Hg or heart rate <50 beats/min (bpm), fetal distress and abdominal pain. The period between the start of uterotonic infusion and labor was 8.44±4.12 h. And the duration of the labor in the patients with prolonged course of labor was 29.15±9.28 h. The most frequently ruptured sites involved isthmus, previous CS line and uterine horns. Persistence for vaginal delivery in patients with a history of CS, cephalopelvic disproportion and prolonged course of labour were presumably the leading causes. In total, 19 patients (31.1%) had a previous history of CS. Total (34.4%) and subtotal (31.1%) abdominal hysterectomies were the most frequent surgical procedures performed. Suture repair was preferred in 34.4% of cases. Hypogastric artery ligation was performed to 18 patients (29.5%). The clinical and procedure related details are demonstrated on Table-II.

The mean operative time was 128.5 minutes (90-180). The average postoperative hospital stay was 8.4 days (4-27). Only two fetal anomalies were detected in this series. All patients needed blood transfusion and mean blood volume transfused was 6.02 Unit. The febrile morbidity rate was 18%. Urinary system injuries were encountered in 14 cases (22.9%). No mortalities have occurred in our UR series. Maternal and fetal outcomes are demonstrated on Table-III.

Correlation analysis of variables revealed that age (rs=0.237, p=0.012) and number of previous pregnancies (rs=0.078, p=0.019) were correlated with the duration of hospital stay after surgery for UR. The delay between UR and surgical intervention was correlated with the duration of hospitalization (rs=0.207, p=0.029) (Table-IV).

## DISCUSSION

Rupture of the gravid uterus is an unexpected and devastating complication of pregnancy with high maternal and fetal mortality and morbidity. Even though it can be prevented in most cases, rates of maternal and perinatal morbidity and mortality are still high.^5^^,^^7^^,^^8^

Modes of presentation in UR may differ in scarred and unscarred uteri: Hypotension and intrauterine death occur frequently in the unscarred UR, whereas abdominal tenderness and fetal distress are more common in the scarred uteri. Rupture of the unscarred uterus carries more hazardous fetomaternal risks compared to scarred uterus.^4^^,^^5^ Even though the mode of presentation was similar for patients with and without CS history, instability, deterioration of vital parameters and vaginal bleeding after labour must remind likelihood of UR.

Hysterectomy -whether total or subtotal- was the main surgical procedure in case of UR. In circumstances where preservation of fertility is an issue to be remembered, suture repair can be considered. However, UR has a potential for mortality and cost-benefit ratio must be evaluated very well. Improved access to resources and services may aid in the avoidance of the vast majority of these mortalities and morbidities.

In the literature, maternal mortality rate can be as high as 13.5%, whereas several other studies from developing countries have reported lower rates.^1^^,^^3^^,^^5^^,^^7^ Even though we did not come across any mortality, deaths may have occurred prior to the admission to the hospital. Hypovolemic shock is claimed to be the main cause of death and rapid transfer of these patients to tertiary care centers is imperative.

A high index of suspicion and quick referral to a well-equipped center may reduce the incidence of this condition. All patients with a history of cesarean section should deliver in hospitals with facilities for surgery and blood transfusion.^8^^,^^9^ Regular antenatal care and meticulous screening of high-risk patients are very important for effective prevention. Family-planning advice to reduce grandmultiparity, improved access to maternal care, decentralization of obstetric services into peripheral units to prevent home deliveries and good supervision during labor can reduce the incidence of UR.^8^^,^^9^

Although Turkey is a developing country, our relatively low UR incidence may be a reﬂection of the high standard of obstetric care and hospital deliveries. In general, patients had adequate antenatal care by trained physicians and they usually delivered at hospital by obstetricians. The impact of defensive medical practice and the decline in traumatic rupture may be the other reasons for our low incidence.^2^^,^^4^ The predisposing factors of UR in developing countries have been demonstrated as: age 31–35, para >3, and poor antenatal care, grandmultiparity, obstetric trauma from prolonged or neglected labour, malpresentation, external and internal podalic version, breech extraction, manual cervical dilatation, and injudicious use of oxytocin, prostaglandins by untrained paramedics and previous unknown corporeal scar.^5^^,^^7^^-^^10^ In our series, the average parity was higher than 4. In addition, we observed that delay of diagnosis for appropriate surgical intervention was correlated with prolongation of hospital stay. Especially, UR encountered in pregnancies at advanced ages may cause longer hospital stays. Therefore, such pregnancies should be more closely and more carefully monitored in terms of potential complications and morbidities.

Close monitoring of maternal and fetal response to uterine stimulants is mandatory to avoid complications of obstructed labour and overuse of uterine stimulants. Application of external force, vacuum forceps and breech extraction are other possible causes of UR.^8^^,^^9^ In our study, external force application was performed in four patients.

The relationship between UR and previous CS is controversial. Various reports have shown that a previously scarred uterus is a major factor which predisposes towards UR.^2^^,^^4^^,^^5^ Cesarean delivery is the most common cause for a scarred uterus. Owing to the increased rates of CS worldwide, the number of women presenting to the labor ward with a scarred uterus is increasing. This fact brings about an increased risk for any maternal morbidity, including UR.^1^^,^^4^^,^^5^^,^^7^^,^^8^ Nineteen of our patients had a history of CS. The reasons of uterine rupture in the patients with a history of CS in the southeastern of Turkey are low socioeconomic status, the long period of transport from villages to the hospital and trial of labor via vaginal route at home to have a chance of more babies. Whereas, the majority of our patients had no history of CS and any other uterine surgery, therefore the role of previous CS in pathophysiology of UR must not be ignored but should not be exaggerated either. 

The choice of the surgical procedure depends upon the type, location and extent of the tear as well as the patient’s condition and desire for future fertility. Total hysterectomy is the operative procedure of choice, unless cardiovascular decompensation necessitates subtotal hysterectomy or simple suture repair and bilateral tubal ligation. Unhealthy tissue remaining after uterine repair may predispose to problems like infection, DIC, abscess formation and haemorrhage. In circumstances where suture repair is undertaken to preserve fertility, the risk of recurrent rupture is always there.^2^^,^^9^^-^^11^ We consider hysterectomy to be the best treatment for patients who have completed their family.

Sudden fetal heart abnormality in laboring patients should be taken as a potential sign of danger. Total abdominal hysterectomy is the routine operative procedure of choice unless cardiovascular decompensation necessitates subtotal abdominal hysterectomy or simple suture repair and bilateral tubal ligation.^11^^,^^12^ The procedure selected in the management of UR must be individualized depending on the patient’s condition and the type, location, and extent of the rupture. Since UR is a life-threatening obstetric hazard, it should always be kept in mind in the care of obstetrics patients. Non-speciﬁc symptoms like epigastralgia and severe vomiting might be critical hints for UR. Gastrointestinal symptoms may increase the abdominal pressure and trigger UR.^9^ With awareness, timely diagnosis, prompt surgical management and neonatal care, rates of maternal and perinatal mortality and morbidity can be reduced.^10^^-^^12^

Assisted breech delivery and malpresentation have been reported to be signiﬁcantly higher among women with UR.^4^ It is interesting that women with hypertensive disorders had higher rates of UR. A logical explanation might be that these women had higher rates of labor induction. An association between hypertensive disorders and other risk factors, such as advanced maternal age and possibly a previous CS, may partially explain the signiﬁcance.^4^

UR in pregnancy is a major obstetric complication that occurs often without warning. This hazardous event should be kept in mind especially in the presence of predisposing factors. Reduction of fetomaternal morbidity and mortality can be achieved via awareness, prompt diagnosis, rapid replacement of blood loss and improved techniques in surgical management and neonatal care. Prevention is more important than management, and regular antenatal care and hospital deliveries may prevent many cases of UR in pregnancy.

**Table-I:** Demographic features of uterine rupture cases

	*Average*	*Range*	*%*
Age	32	20-45	-
No. of pregnancies	5.2	1-12	-
No. of parities	4.4	0-11	-
Gestational weeks	35.1	20-40	-
Previous CS	19	-	31.1

(*Abbreviation:* CS= Cesarean section)

**Table-II:** Details regarding the etiology and management of uterine rupture

		*No. of cases*	*%*
Causes	Persistence for vaginal delivery after CS	19	31.1
	Cephalopelvic disproportion	11	18
	Prolonged course of labour	8	13.1
	Injudicious use of uterotonics	7	11.5
	Malpresentation	5	8.3
	Application of external force (Kristeller maneuver)	4	6.6
	Partum precipitates	1	1.6
	External abdominal trauma	1	1.6
	Unknown	5	8.2
Symptom	Vaginal bleeding	27	44.3
	Hemodynamic instability	18	29.5
	Fetal distress	7	11.5
	Abdominal pain	6	9.8
Site of rupture	Isthmus	24	39.3
	Previous CS line	19	31.1
	Uterine horn	18	29.6
Type of intervention	TAH	21	34.4
	Suture repair	21	34.4
	SAH	19	31.1

(*Abbreviations:* TAH= total abdominal hysterectomy, SAH= subtotal abdominal hysterectomy.)

**Table-III:** Maternal and fetal outcomes

		*No. of cases*	*%*
Maternal	Uterine atony	19	31.1
	Vesicouterine rupture	8	13.1
	Blood transfusion	61	100
	Relaparataromy due to intraabdominal hemorrhage	7	11.5
	Febrile morbidity	11	18
	Wound infection and dehiscence	5	8.2
	Acute renal failure	2	3.2
	DIC	6	9.8
	Ureter injury	4	6.6
	Uretra injury	1	1.6
	ARDS	1	1.6
	Mortality	0	0
Fetal	Apgar score 1.min	5.22±1.76*	-
	Apgar score 5.min	7.56±1.42*	-
	Fetal birth weight (g)	2940.86±1011.63**	-
	Mortality	25	41
Site of rupture	Isthmus	24	39.3
	Previous CS line	19	31.1
	Uterine horn	18	29.6
Type of intervention	TAH	21	34.4
	Suture repair	21	34.4
	SAH	19	31.1

*(Abbreviations:* DIC =disseminated intravascular coagulation, ARDS=Acute respiratory

distress syndrome, *=mean±standart deviation (SD) values in live borns, **mean±SD )

**Table-IV:** Correlation analysis of variables correlated with the duration of hospitalization

*Correlations*	*r*		*p Value*
Age * Duration of hospitalization	0.237		0.012
Number of previous pregnancies* Duration of hospitalization	0.078		0.019
Surgical delay * Duration of hospitalization	0.207	0.029	

(Kendall's tau-b)

In conclusion, UR constitutes a major risk factor for fetomaternal morbidity and mortality. A high index of suspicion for UR must be reserved for women presenting with evidence of hypovolemia and fetal compromise. Early diagnosis and prompt recourse to exploratory laparotomy can be necessary to save the fetus and avoid further complications. Increased accessibility to antenatal care as well as a functional referral system and popularization of information, education and communication programmes for pregnant women may aid in prevention of UR.

## Authors Contribution

Abdulkadir Turgut, Ali Ozler: Designed the research protocol and literature search.

Abdulkadir Turgut, Mehmet Siddik Evsen, Hatice Ender Soydinc, Neval Yaman Goruk, Talip Karacor: Conducted the study and did data analysis.

Abdulkadir Turgut, Talip Gul: Prepared the final draft for publication.
